# Altered Expression of Neuroplasticity-Related Genes in Alcohol Addiction and Treatment

**DOI:** 10.3390/ijms252111349

**Published:** 2024-10-22

**Authors:** Evangelia Legaki, Nikolas Dovrolis, Nikoletta Moscholiou, Ilias Koutromanos, Efthimios Vassilopoulos, Antonios Dakanalis, Maria Gazouli, Elias Tzavellas

**Affiliations:** 1 Laboratory of Biology, Department of Basic Biological Science, School of Medicine, National and Kapodistrian University of Athens, 11527 Athens, Greece; evaglegaki@med.uoa.gr (E.L.); ndovroli@med.uoa.gr (N.D.); nikoletta.moscholiou@gmail.com (N.M.); 2 First Department of Psychiatry, “Aiginition” Hospital, School of Medicine, National and Kapodistrian University of Athens, 11528 Athens, Greece; koutro.ilias.23@gmail.com (I.K.); efvasilop@med.uoa.gr (E.V.); 3 Department of Psychiatry and Psychotherapy, Psychiatric Services Aargou AG, 5210 Brugg-Windisch, Switzerland; 4 Department of Medicine and Surgery, University of Milano Bicocca, Via Cadore 38, 20900 Monza, Italy; antonios.dakanalis@unimib.it

**Keywords:** alcohol use disorder (AUD), synaptic plasticity, neuroplasticity, gene expression, biomarker

## Abstract

Alcohol use disorder’s complexity arises from genetic and environmental factors, with alcohol metabolism genes and neurotransmitter pathways being critical. This study aims to analyze synaptic plasticity gene expression changes in individuals with AUD in order to study their contribution to AUD development and to identify potential biomarkers of treatment response. RNA was extracted from whole peripheral blood (20 patients, 10 healthy controls), before and after treatment (Qiagen AllPrep RNA/DNA Mini Kit), and the gene expression of 84 genes related to neuroplasticity was studied using the RT2 Profiler for Human Synaptic Plasticity RT-PCR Array (PAHS-126ZA, Qiagen), comparing AUD patients to control and responders to non-responders. The potential prognostic/predictive biomarkers were searched using machine learning models. A total of 35 dysregulated genes were found in AUD patients. *EPHB2*, *EGR*, and *AKT1* were increased, while *TIMP1*, *NCAM1*, and *GRM2* were decreased. Responders showed distinct gene expression profiles at baseline. After treatment, the expression of 57 genes was normalized, while *NCAM1*, *GRM2*, and *BDNF* showed the most significant recovery. *EGR4*, *INHBA*, and *NCAM1* emerged as potential biomarkers to predict treatment success. These results indicate that gene profiles in peripheral blood can serve as prognostic markers for the prognosis and treatment of AUD, although further validation is required.

## 1. Introduction

Alcohol use disorder (AUD) is a significant global public health challenge affecting millions of people. Defined by excessive drinking and persistent alcohol-seeking behavior, AUD encompasses a spectrum of problematic use leading to substantial impairment, as outlined in the Diagnostic and Statistical Manual of Mental Disorders (DSM-V) [1]. The disorder affects nearly 6% of individuals annually, contributing to profound socio-economic and public health burdens worldwide [2]. According to WHO data from 2016, alcohol abuse rates among males in Greece were reported at 9.4%, with females at 2.9%. Meanwhile, alcohol dependence affected 4.2% of males and 1.3% of females, suggesting lower prevalence rates compared to Northern Europe [3]. Over the past two decades, national trends indicate a reduction in overall alcohol consumption in Greece; however, alcohol consumption among Greek teenagers remains among the highest in Europe [4].

While the physical health implications of AUD are well documented, its profound impact on mental health remains a critical concern. The disorder’s pathophysiology is complex, involving genetic predisposition, environmental factors, and cumulative effects of alcohol consumption, underscoring the need for effective treatment strategies [5]. Genome-wide association studies (GWAS) have revealed significant insights into how specific genes contribute to susceptibility and severity of AUD. Key genes involved in the breakdown of alcohol, such as alcohol dehydrogenases (ADH) and aldehyde dehydrogenase (ALDH), play essential roles. In addition to alcohol metabolism, genes related to neurotransmitter systems are also implicated. Serotonin and dopamine pathways, crucial for regulating mood and processing rewards, have been associated with susceptibility to alcohol use disorder (AUD). Variations in genes affecting these systems can impact neurotransmitter levels, potentially influencing an individual’s inclination to seek out and respond to alcohol. Furthermore, genes within the Dynorphin Kappa Opioid Receptor System, the Endocannabinoid system, and pathways involved in synaptic plasticity also contribute to AUD. These systems regulate brain functions such as stress response, reward mechanisms, and neuronal communication, all of which are critical in the onset and progression of AUD [6].

Synaptic plasticity is the property of synapses to strengthen or weaken in response to changes in both the amplitude and the temporal dynamics of neuronal activity. Sensory inputs and intrinsic brain activity can affect long-term changes in synaptic efficacy and eventually increase or decrease neuronal connectivity by modulating the number of synapses [7,8]. Synaptic plasticity, essential for brain development and function, enables adaptability, learning, and overall mental well-being [9]. This critical brain function also plays a key role in both short- and long-term memory, and the mechanisms underlying these changes have been linked to the pathophysiology and treatment of multiple neurobiological disorders, including depression [10]. Synaptic plasticity is also crucial in the early development of neural circuitry, and evidence is accumulating that impairments in synaptic plasticity mechanisms contribute to several prominent neuropsychiatric disorders [7].

Environmental factors such as stress, trauma, and substance use can profoundly affect synaptic plasticity, contributing to a range of psychiatric disorders. Unlike focal brain injuries, these disorders involve widespread changes across brain regions crucial for cognition, emotion regulation, and motivation. They are influenced by complex genetic factors shaped by both neurodevelopment and environmental experiences, leading to chronic, recurrent conditions with slow recovery and high relapse rates. Genetic investigations suggest the involvement of genes like brain-derived neurotrophic factor (BDNF) and the NMDA receptor in these disorders, influencing synaptic connectivity [9].

Likewise, repeated substance use results in neurobiological changes, which alters the brain’s reward circuitry and increases synaptic plasticity in brain areas such as the prefrontal cortex, amygdala, and striatum. These changes heighten motivation for the substance, leading to tolerance, dependence, and withdrawal symptoms. By understanding these dysregulations in synaptic plasticity, we gain valuable insights into developing targeted therapies for psychiatric disorders [9].

Concerning the AUD therapeutic approaches, individuals who are struggling with alcohol dependence can receive assistance from the special inpatient alcohol detoxification units of the hospitals by taking part in any one of a comprehensive range of evidence-based treatment options.

Taken the above into consideration, in this study, we aimed to profile gene expression changes associated with alcohol abuse and to identify specific baseline differences between individuals who will and will not respond to therapeutic interventions. By comparing the transcriptomic profiles of these individuals before and after the intervention, we aim to quantify the effects of therapeutic intervention on transcription levels. This investigation will facilitate the differentiation of patient groups based on established gene expression profiles, thereby advancing both clinical and research applications in the context of therapeutic interventions for alcohol use disorder.

## 2. Results

### 2.1. Effect of Alcohol Abuse on Synaptic Plasticity

**Figure 1:**
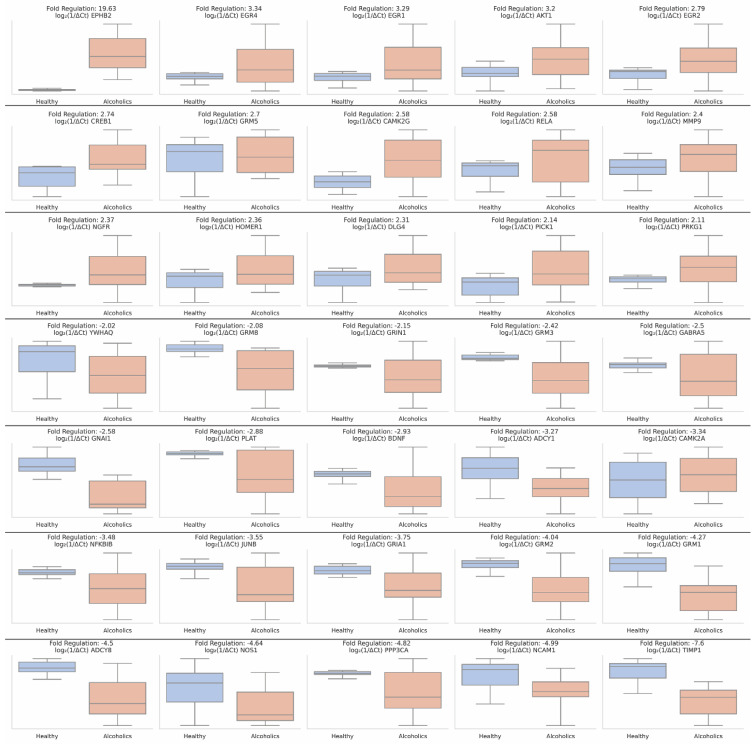
Differential expression of synaptic plasticity-related genes in alcohol use disorder (AUD) versus healthy controls. Box plots representing the fold regulation of 35 synaptic plasticity-related genes in AUD patients compared to healthy controls. Gene expression was transformed to log_2_(1/ΔCt) values for visualization.

As previously described, we combined the samples of alcoholics at baseline, regardless of their response to treatment later and compared them with healthy controls. Statistically significant changes (*p* < 0.05 and fold regulation of ±2) highlight, in total, 35 dysregulated genes. *EPHB2* showed the highest positive fold regulation of 19.63, indicating substantial upregulation. Three EGR family members, *EGR4*, *EGR1*, and *EGR2* exhibited a notable upregulation with fold changes of 3.34, 3.29, and 2.79, respectively. *AKT1* was upregulated with a fold change of 3.2, and *CREB1* had an upregulation of 2.74-fold. *GRM5* was upregulated with a fold change of 2.7, while *CAMK2G* and *RELA* both showed a fold regulation of 2.58. *MMP9* exhibited an upregulation of 2.4 fold, *NGFR* of 2.37, and *HOMER1* of 2.36-fold. Finally, *DLG4* was upregulated with a fold change of 2.31, *PICK1* of 2.14, and *PRKG1* 2.11 fold. On the other end, the genes *YWHAQ*, *GRM8*, *GRIN1*, *GRM3*, *GABRA5*, and *GNAI1* were moderately downregulated with a fold regulation of −2.02, −2.08, −2.15, −2.42, −2.5, −2.58, respectively. *PLAT* had a downregulation of −2.88, *BDNF* −2.93, and *ADCY1* of −3.27 fold. *CAMK2A* exhibited a −3.34 fold downregulation while *NFKBIB* demonstrated a fold regulation of −3.48 fold. *JUNB* was downregulated −3.55 fold, *GRIA1* −3.75 fold, *GRM2* −4.04 fold, *GRM1* −4.27 fold, *ADCY8* −4.5 fold, *NOS1* −4.64 fold, *PPP3CA* −4.82 fold, and *NCAM1* showed a fold regulation of −4.99 fold. *TIMP1* had the highest negative fold regulation of −7.6, indicating a substantial downregulation (Figure 1).

**Figure 2:**
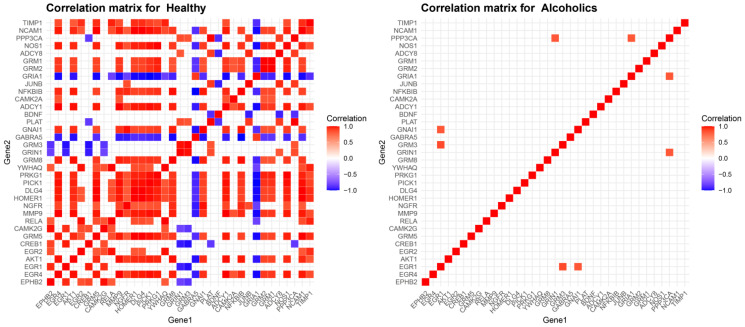
Co-expression analysis of synaptic plasticity-related genes in healthy controls and alcoholics. Spearman correlation heatmaps for synaptic plasticity-related genes in healthy controls (**left**) and alcoholics (**right**). Results filtered for rho less than −0.8 and greater than 0.8.

Studying the correlations between these dysregulated genes to identify co-expression patterns in both the alcoholic and control groups allows us to discern specific patterns indicating synergistic roles or the presence of common, invisible for this analysis, transcriptional factors. To highlight the strongest gene pairwise correlations, as described in our methodology, we focused on Spearman correlation coefficients higher than 0.8 for strong positive and lower than −0.8 for strong negative correlations (Figure 2).

The healthy control group showed a remarkably greater presence of such correlations indicative of high synaptic activity and plasticity. For example, in the healthy control group *EGR4*, *MMP9*, *ADCY1*, *AKT1*, and PICK1 paired with each other with correlations of 1.00 and 0.99, revealing a strongly co-regulated cluster. The same pattern is shown for *HOMER1*, *GRM5*, *DLG4*, *NCAM1*, and *PICK1*, with the same rho values. Interestingly *GRIA1* shows strong negative correlations with multiple of these genes, such as *EGR4*, *GRM5*, *MMP9*, *HOMER1*, *DLG4*, *PRKG1*, *ADCY1*, *NOS1*, *NCAM1*, and *PICK1*. The results of the alcoholic group are significantly less showing of no inverse correlation between these genes, showing no pairwise correlation of rho 0.90–1.00 and only highlighting the pairs of *GRM3* and *EGR1* with the highest correlation with a coefficient of 0.86, *EGR1* and *GNAI1* with a coefficient of 0.82, *PPP3CA* and *GRIA1* with a coefficient of 0.82, and finally *PPP3CA* and *GRIN1*, which share a correlation coefficient of 0.82. Interestingly, even though the genes *GRM3* and *GNAI1* or *GRIA1* and *GRIN1* share correlations with the genes *EGR1* and *PPP3CA*, respectively, they could not surpass our rho > 0.8 threshold as pairs.

### 2.2. Prediction of Response to Clinical Therapeutic Intervention

Prediction of response to therapeutic intervention is a cornerstone of precision medicine. To identify with a simple molecular technique if a patient will be more susceptible to a specific intervention before starting is crucial in deciding the next steps in therapy. For that reason, as described in our methodology, we decided to compare the transcriptomic profiles of patients who will or will not respond to our intervention versus each other. This comparison allows, in a clinical and a research setting, us to differentiate the different groups based on already established profiles.

**Figure 3:**
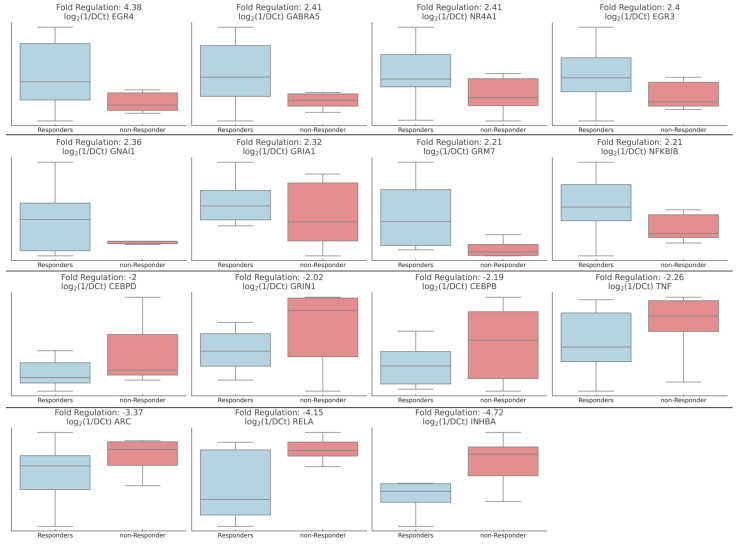
Baseline gene expression differences between responders and non-responders to alcohol detoxification therapy. Box plots representing the fold regulation of 15 synaptic plasticity-related genes. Gene expression was transformed to log_2_(1/ΔCt) values for visualization. These gene expression profiles suggest potential biomarkers that could predict therapeutic response in AUD patients.

In total, 15 genes showed significant differences when comparing responders to non-responders to intervention at baseline. *EGR4* exhibits the highest upregulation with a fold regulation of 4.38, indicating a strong biomarker candidate. This result is followed by *GABRA5* and *NR4A1*, both with a fold regulation of 2.41. *EGR3* is upregulated with a fold regulation of 2.4, while *GNAI1* shows an increase at 2.36, *GRIA1* is upregulated by 2.32-fold, and both *GRM7* and *NFKBIB* exhibit a fold regulation of 2.21. On the other hand, some genes are downregulated, with *INHBA* showing the most substantial decrease at −4.72, followed by *RELA* at −4.15, while *ARC* has a fold regulation of −3.37, and *TNF* is downregulated by −2.26 fold. *CEBPB* and *CEBPD* are downregulated by −2.19 and −2-fold, respectively. Finally, *GRIN1* shows the least downregulation at −2.02 (Figure 3).

**Figure 4:**
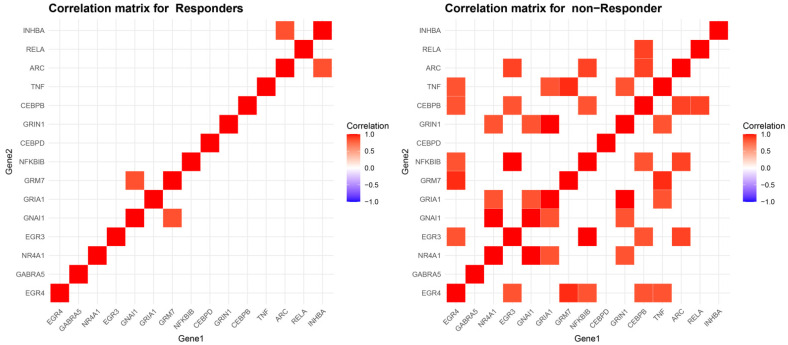
Correlation matrix of synaptic plasticity-related genes in responders versus non-responders to alcohol detoxification therapy. Spearman correlation heatmaps for synaptic plasticity-related genes in responders (**left**) and non-responders (**right**) to therapy. Responders show limited co-expression between gene pairs, while non-responders exhibit a more extensive and complex gene interaction network, suggesting that non-responders may have more widespread dysregulation in synaptic plasticity-related pathways. Results filtered for rho less than −0.8 and greater than 0.8.

Examining the co-expression of these genes in our two groups individually, we see that, in the responder group, only the pairs of *GRM7*-*GNAI1* and *INHBA*-*ARC* are strongly correlated, both with a rho of 0.84, while, in non-responders, the correlation network is much more expansive. Notably, in non-responders, the pairs of *GNAI1*-*NR4A1*, *NFKBIB*-*EGR3*, and *GRIA1*-*GRIN1* exhibit an absolute correlation coefficient of 1.00, while *GRM7*-*EGR4* and *TNF*-*GRM7* show a rho of 0.94, among others (Figure 4).

### 2.3. Effect of Therapeutic Intervention on Transcriptomic Profile

**Figure 5:**
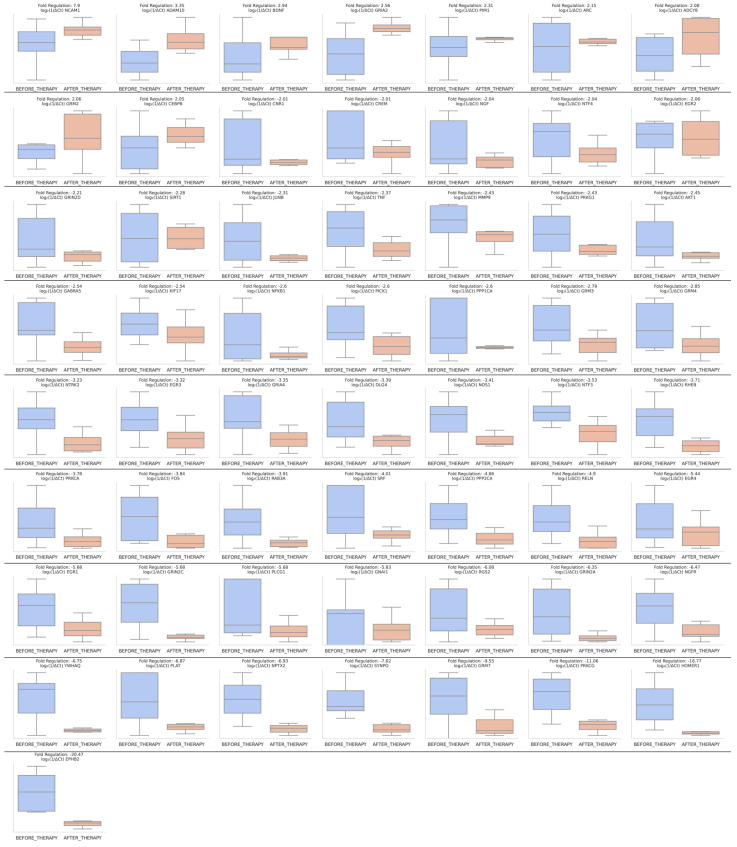
Gene expression changes before and after alcohol detoxification therapy in responders. Box plots representing the fold regulation of 57 synaptic plasticity-related genes. Gene expression was transformed to log_2_(1/ΔCt) values for visualization. Previously upregulated genes such as *EGR4*, EPHB2, and HOMER1 are downregulated after therapy, suggesting a reversal of alcohol-induced dysregulation.

Quantifying response to a treatment requires a combination of clinical observations and molecular markers. To quantify the effects of our therapeutic intervention, we are investigating the effects on transcription levels before and after the therapeutic intervention. DGEA highlights a significant reversal of the effects of alcohol on gene expression, presenting most of the genes previously dysregulated, striving to match healthy control levels with a particular emphasis on downregulating gene expression previously exacerbated by alcohol. In total, 57 genes showed differential expression after the intervention, 18 of whom were previously found dysregulated due to alcohol (Figure 5).

In particular, *NCAM1* exhibited a significant upregulation by 7.9-fold (previously downregulated in alcoholics versus healthy controls by −5.53 fold), *ADCY8* showed an upregulation with a fold change of 2.08 (previously −3.04 fold), *GRM2* showed a 2.06-fold increase (previously −3.57 fold), and *BDNF* was upregulated by 2.94-fold (previously downregulated in alcoholics versus healthy controls by −3.32 fold). Similarly, several genes demonstrated downregulation after treatment on the responders. Specifically, *MMP9* showed a fold change of −2.43 (previously upregulated in alcoholics versus healthy controls by 2.03-fold), *AKT1* demonstrated a fold regulation of −2.45 (previously upregulated by 3.25-fold), *EGR2* was downregulated by −2.06-fold (previously upregulated by 2.49-fold), and *DLG4* showed a fold change of −3.39 (previously upregulated by 2.16-fold). *NGFR* demonstrated a fold regulation of −6.47-fold (previously upregulated in alcoholics versus healthy controls by 2.33-fold), *RELN* exhibited a fold change of −4.9 (previously upregulated by 2.03-fold), *EGR1* demonstrated a fold change of −5.68 (previously upregulated by 3.68-fold), while *EGR4* exhibited a fold change of −5.44 (previously upregulated in alcoholics versus healthy controls by 5.2-fold). The most substantial downregulations were observed in *HOMER1* with a fold change of −16.77 and *EPHB2* with a fold change of −20.47, both previously upregulated in alcoholics versus healthy controls by 2.78 and 20.81-fold, respectively. Interestingly, *JUNB* exhibited a downregulation of −2.31 fold, NOS1 of −3.41, *YWHAQ* −6.75-fold, and *PLAT* −6.87-fold, further downregulating genes whose expression was negatively impacted by alcohol versus healthy controls.

**Figure 6:**
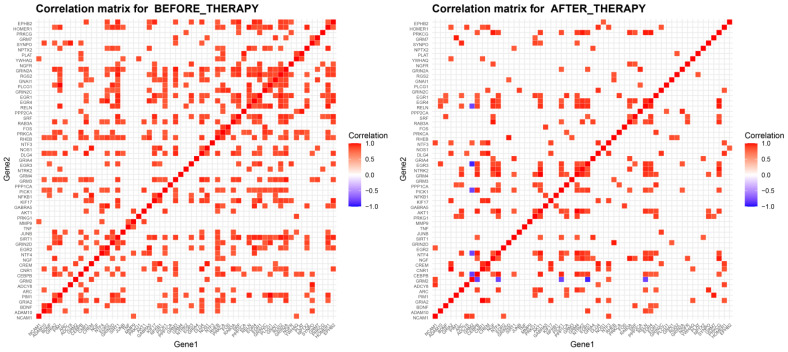
Correlation matrix of synaptic plasticity-related genes before and after alcohol detoxification therapy in responders. Spearman correlation heatmaps for synaptic plasticity-related genes in responders before (**left**) and after (**right**) therapy. The therapy appears to restore the balance in synaptic plasticity-related gene expression, as evidenced by the reduction in co-expression patterns. Results filtered for rho less than −0.8 and greater than 0.8.

Regarding co-expression of the 57 perturbed genes in which we observed high activity before and after intervention but with significant changes in their correlation profiles, after therapy, we had almost half the correlations achieving a rho less than −0.8 and greater than 0.8 (196 after vs. 355 before), reminiscent of the lower activity of the healthy controls (Figure 6).

In addition, we noticed no significant inverse correlation in alcoholics before treatment for these genes, but after treatment, the pair of *GRM2*-*EGR3* had a rho of −0.93, the pair *GRM2*-*CEBPB* of −0.83, the pair *GRM2*-*PICK1* of −0.81, and the pair *GRM2*-*NTF4* of −0.81.

## 3. Discussion

This study focuses on how alcohol addiction directly influences the expression levels of blood cell neuroplasticity-related genes in humans. In addition, it allows us to identify how synaptic plasticity can be targeted by therapeutic intervention restoring the “normal” transcriptomic profile. As previously suggested, the blood cell and CNS correlation may seem logical if one considers that blood cells regularly travel through many different body regions and may be exposed to the same environment as CNS tissue; having relatively direct access to the blood, they detect and respond to its changes [11]. It is well accepted that blood-based biomarkers are increasingly recognized for their utility and relevance in psychiatric disorders, as they provide quantifiable insights into the biological underpinnings of mental health conditions. By integrating these biomarkers into the biopsychosocial model, researchers can better understand the complex interactions between biological, psychological, and social factors that contribute to psychiatric disorders. This knowledge enhances diagnostic accuracy, facilitates early intervention, and enables personalized treatment strategies tailored to individual patient needs. Furthermore, blood biomarkers have the potential to illuminate the pathophysiology of mental health conditions, ultimately improving patient outcomes and reducing the global burden of these disorders [12,13,14].

Our study reveals 35 dysregulated genes that can effectively differentiate between alcoholics and healthy controls. Among them, most notably, *EPHB2* was found to be significantly upregulated to alcoholics. It is well known that *EPHB2* deficiency has been related to depression-like conditions and memory impairments in animal studies [15]. Interestingly, in accordance with our findings, Sanz-Martos et al. [16] observed increased expression of *EPHB2* in ethanol-exposed rats. The role of the EphB2 protein is the signal transmission in hippocampal development, and it also plays a basic role in synapse formation and synaptic stabilization [17,18]. Several studies supported that, since EPHB2 is implicated in synaptic plasticity [19] and considering the interaction with alcohol [20], it can be an attractive target for future studies.

The EGR family genes, including *EGR1*, *EGR2*, and *EGR4*, have shown significant upregulation in the alcoholic group of the present study. The EGR genes encode a family of immediate early gene transcription factors that mediate the transcription of various genes related to neuronal development and plasticity, cognition, circadian rhythm, and social behaviors [21]. This family of genes has been referred to be implicated in various psychiatric conditions. For instance, *EGR1* has been closely associated with major depressive disorder (MDD). Stress-related fear memory has been linked to increased *EGR1* expression, and knocking down *EGR1* expression effectively blocks the fear-related response [22]. Reversely, in depression and schizophrenia, most studies revealed a decreased expression of EGR genes. Covington et al. (2010) observed a decreased expression of *EGR1* in the medial prefrontal cortex of depressed patients who were refractory to treatment, as well as in non-medicated subjects. Notably, this brain region was consistently reported as affected in both human patients and animal models of depression [23]. Moreover, the downregulation of *EGR1*, *EGR2*, and *EGR3* transcripts was reported in the postmortem brains of patients with schizophrenia [24]. Among EGR genes, *EGR3* has demonstrated a strong and consistent association with schizophrenia in both family-based and case–control association studies within Japan [24] and Korea [25]. Additionally, a genetic link between *EGR2* and *EGR3* and bipolar disorder was recently identified [26]. However, our results did not reveal a significant differential expression of *EGR3*, similar to a study conducted in a Chinese population that reported no association [27].

Our study also highlighted the upregulation of *AKT1*; however, to the best of our knowledge, there are scarce data about the association of *AKT1* with alcohol disorder or other psychiatric conditions. Excessive alcohol intake has been shown to result in a sustained activation of the AKT pathway but not the *ERK1/2* pathway in the nucleus accumbens (NAc) [28]. This activation is brain region-specific and is believed to engage the *AKT/mTORC1* pathway, contributing to mechanisms in the NAc shell and orbitofrontal cortex (OFC) that play a key role in the development and persistence of alcohol-drinking behaviors. In contrast, decreased *AKT1* expression was observed in the dorsolateral prefrontal cortex of individuals with schizophrenia and bipolar disorder, with a significant reduction in schizophrenic patients and a moderate effect size in bipolar patients [29]. These findings underline the distinct involvement of the AKT pathway in both alcohol-related behavior and psychiatric disorders.

Furthermore, our results identified the upregulation of the gene *CREB1* in individuals with alcohol dependence compared to healthy controls. *CREB1* encodes the cyclic AMP-responsive element-binding protein–1 (*CREB1*), a transcription factor crucial for neuronal activity, synaptic plasticity, learning, and memory processes, including semantic memory, episodic memory, and executive functions [30,31,32,33]. CREB signaling, which is regulated through CREB phosphorylation, plays a significant role in neural plasticity and is disrupted in various pathological states, such as addiction and psychiatric disorders, including depression and schizophrenia [34,35]. The phosphorylation of CREB leads to its nuclear localization and recruitment of transcriptional coactivators, such as the CREB binding protein (CBP) and p300. These coactivators possess intrinsic histone acetyltransferase (HAT) activity, catalyzing histone acetylation—a key epigenetic modification associated with increased gene transcription [36]. The dysregulation of CREB signaling, particularly in the context of addiction, is thought to alter synaptic plasticity through mechanisms like long-term potentiation (LTP) and long-term depression (LTD), which are essential for the development of compulsive behaviors and addiction progression [37,38]. Several studies explored the role of *CREB1* in psychiatric conditions, notably schizophrenia. On a genetic level, *CREB1* polymorphisms have been linked to schizophrenia, bipolar disorder, and major depressive disorder [39,40,41]. However, transcriptomic analyses produced conflicting results regarding *CREB1* expression in schizophrenia. While some studies report downregulation in the prefrontal cortex (PFC) [41], others, including a meta-analysis by Ohayon et al. (2020), have found upregulation in Brodmann Area 10 (BA10) [42] and the dorsolateral prefrontal cortex (DLPFC) [43]. This discrepancy might be attributed to differences in brain regions studied or methodological variations. Interestingly, animal models suggest that elevated CREB expression could lead to reduced sensitivity to emotional stimuli, anhedonia, and depressive-like behaviors [44,45,46,47,48], further emphasizing *CREB1*’s complex role in mental health disorders. Moreover, CREB1 regulates several genes involved in synaptic plasticity, including *BDNF* together with several glutamatergic genes (*GRM5*, *GRM7*, *GRID1*, and *GRIN2A*) and protein kinase C genes (*PRKCA* and *PRKCB*,) underscoring its pivotal role in modulating the neural circuits that underlie learning, memory, and potentially addiction [49].

Recent research into the role of metabotropic glutamate receptors (mGluRs) in alcohol use disorder (AUD) has uncovered significant insights into how these receptors influence alcohol-related behaviors. The metabotropic glutamate (mGlu) receptor family, consisting of eight subtypes (*mGlu1* to *mGlu8*), is divided into three groups based on their genetic, pharmacological, and functional characteristics. Group I receptors (*mGlu1* and *mGlu5*) are primarily located at postsynaptic sites, where they signal through Gq proteins, leading to the activation of pathways involved in synaptic plasticity and neuronal excitability. In contrast, Group II (*mGlu2* and *mGlu3*) and Group III (*mGlu4*, *mGlu6*–*mGlu8*) receptors are mainly found at presynaptic locations and couple with Gi proteins to inhibit neurotransmitter release by reducing cyclic AMP (cAMP) levels [50]. Our data revealed an increased expression of *GRM5* and a decreased expression of *GRM1* and *GRM2* in blood samples from individuals with AUD when compared to healthy controls. Our findings of increased *GRM5* expression in AUD patients is consistent with the existing literature that suggests *GRM5* plays a critical role in the modulation of alcohol-related behaviors [51,52,53]. Studies have shown that *mGluR5* is involved in various aspects of mood disorders and addiction [54], including the reinforcement of alcohol consumption and the regulation of negative affect associated with AUD [55]. For instance, Kasten et al. (2019) highlighted that modulation of *mGluR5* could reduce ethanol intake and alleviate alcohol-induced anxiety-like behavior, emphasizing its potential as a therapeutic target. This receptor’s upregulation may serve as a compensatory response to prolonged alcohol exposure [55]. This response aligns with rodent studies, where inhibiting or genetically targeting *GRM5* was shown to significantly reduce alcohol self-administration and relapse-like behavior [56]. However, this receptor’s role is complex, with some studies noting variability in its expression depending on the brain region, the type of alcohol exposure, and the time since the last exposure [51]. This complexity might explain the increased *GRM5* expression observed in our study, reflecting its dynamic regulation in response to chronic alcohol use.

Conversely, our data showed a decrease in *GRM1* and *GRM2* expression in blood samples from individuals with AUD, which diverges from some aspects of the existing literature. While the role of *GRM1* in AUD is well documented, with studies indicating that *mGlu1*, along with *mGlu5*, is involved in the glutamatergic system’s regulation of alcohol-related behaviors, the literature often reports an upregulation of *mGluR1* in specific brain regions of animal models (nucleus accumbens, central amygdala) in response to alcohol exposure [57,58,59,60,61,62]. However, other studies highlighted that chronic alcohol exposure was shown to decrease *mGlu1* mRNA expression in brain regions such as the hippocampus and cerebellum [63]. The reduced expression of *GRM1* in our study might reflect similar mechanisms in peripheral tissues with some brain regions, potentially serving as a marker for the neuroadaptive changes occurring centrally or indicating a compensatory response to chronic alcohol exposure. This reduction is associated with increased sensitivity to the neurochemical effects of alcohol, supporting the notion that diminished *mGlu1* function may exacerbate alcohol-related behaviors [64,65,66].

The decrease in *GRM2* expression is particularly noteworthy given that *GRM2* encodes *mGluR2*, a receptor critical for synaptic transmission and the regulation of the alcohol-related reward system. Previous research has demonstrated that loss or reduction in *mGluR2* function leads to insufficient control of glutamate release, contributing to excessive alcohol consumption [67]. Our findings are consistent with these observations, suggesting that reduced *GRM2* expression may be a key factor in the dysregulation of glutamatergic signaling in AUD, particularly in key brain regions like the nucleus accumbens and the ventral tegmental area [50].

The *HOMER1* gene, identified as upregulated in our study, plays a crucial role in the glutamatergic pathway, primarily through its interactions with Group I metabotropic glutamate receptors (mGluRs), specifically *mGlu1* and *mGlu5*. Homer proteins, including those encoded by *HOMER1*, are essential for regulating behavioral sensitivity to alcohol and other drugs of abuse. Among the postsynaptic scaffolding proteins, the Homer family has been well characterized, particularly as a key element influencing synaptic plasticity [68]. Given that *GRM5* (encoding mGluR5) was also upregulated in our study, it is plausible that increased *HOMER1* expression similarly facilitates *mGluR5* activity, thereby contributing to alcohol’s reinforcing effects. A prior study examined the association between genetic variants of *HOMER1* and *HOMER2*, located on chromosomes 5q14.2 and 15q24.3, respectively, with cocaine dependence. This study reported a significant link between the *HOMER1* SNP rs6871510 genotypes and cocaine dependence [69].

While there are limited data on *HOMER1* expression in alcohol use disorder (AUD), most findings focus on *HOMER2*. Research, both preclinical and clinical, has underscored the significance of *HOMER2* isoforms as active contributors to alcohol-induced behavioral and cellular plasticity [70,71]. Overexpression of *HOMER2* is linked to an increase in alcohol’s potency and efficacy in eliciting reward, through its enhancement of both appetitive and consummatory behaviors related to alcohol. This upregulation likely enhances alcohol’s rewarding effects by promoting *mGlu1*/5 interactions. In contrast, *HOMER2* deletion, knockdown, or blockade results in an alcohol-avoiding phenotype [58,60,71,72,73].

Interestingly, our data also revealed an increased expression of *CAMK2G* and a decreased expression of *GRIA1*. The current literature lacks enough data for the association of *CAMK2G* and alcohol disorder. CaMKII, particularly its α-subunit (αCaMKII), plays a key role in learning, memory, and addiction. Most data have shown *CAMK2A* involvement in drug addiction in both humans and animal models. *CaMKII* levels in the ventral tegmental area (VTA) and nucleus accumbens (NAcc) are critical for psychostimulant sensitization and self-administration. Though less is known about its role in alcohol addiction, alcohol has been found to affect brain CaMKII levels and increase phosphorylation at *Thr286*, influencing cellular signaling, behavior, and neurotoxicity [74]. Conversely, the downregulation of *GRIA1*, which encodes the *AMPA* receptor subunit 1, is particularly important. *GRIA1* is crucial for fast excitatory synaptic transmission, and its reduced expression may impair *AMPA* receptor-mediated signaling, potentially disrupting synaptic plasticity necessary for learning and memory. This aligns with studies showing decreased *GRIA1* and *GRIA2* transcript levels in the medial prefrontal cortex (mPFC) of alcohol-dependent mice, suggesting a role for *AMPAR* plasticity in the glutamatergic dysfunction associated with ethanol withdrawal. A negative correlation between *GRIA1* mRNA levels and total ethanol intake further suggests that reduced *GRIA1* expression may be linked to higher alcohol consumption, reinforcing the idea that ethanol exposure disrupts *AMPA* receptor function and contributes to addiction-related neural changes [75]. Notably, our findings are further supported by prior research that used a pathway-based approach to examine genetic variants within this system. Variants in genes such as *GRM1*, *GRM5*, *HOMER1*, *HOMER2*, *EIF4E*, *EEF2*, *GRIA1*, *GRIA4*, *MTOR*, and *CAMK2A* have been shown to be associated with increased alcohol consumption, specifically the number of drinking days per month, when considered collectively. Furthermore, hypermethylation at a CpG site in the 3′-untranslated region of *EEF2* was observed in individuals who consumed alcohol more frequently, indicating potential epigenetic regulation of this pathway in relation to alcohol use [66].

Furthermore, *TIMP-1* was downregulated while *MMP9* levels were elevated in subjects with alcohol intoxication in our study. Acute alcohol intoxication has been associated with increased cytokine production, oxidative stress, and liver apoptosis [76]. Recent research suggests that even a single binge drinking episode can disturb the balance of the fibrosis process [77]. Zdanowicz et al. suggest that even one episode of alcohol intoxication in adolescents can disrupt fibrosis markers, with *MMP9* and *TIMP-1* emerging as more sensitive indicators of liver damage than ALT and AST. While *MMP9* and *TIMP-1* are expressed in various organs, the elevated levels observed in this study likely reflect the direct impact of ethanol on the liver [78]. Additionally, *MMP9* polymorphisms, such as the C/T variant, have been linked to alcohol dependence and elevated serum *MMP9* levels [79]. In *MMP9* knockout mice, chronic alcohol exposure impaired synaptic plasticity and reduced alcohol-seeking behavior, both of which were restored by reintroducing *MMP9* [80]. This same polymorphism has also been associated with increased motivation to drink in alcoholic patients.

Limitations of our study include the relatively small number of samples, which, combined with our targeted approach, cannot produce prediction models capable of generalization. In addition, confounding factors, like smoking [81], can influence gene expression profiles in blood samples and should be considered. In our study, however, due to the high accuracy provided by strong differential gene expression in our limited samples, those factors only add complexity to the models without having any real impact on their accuracy. Regardless, the results of this study further strengthen the value of expression data in predicting response to alcohol detoxification intervention and its effects. At baseline, 15 genes were identified with significant differences in expression, helping classify the samples and positioning genes such as *EGR4*, *GABRA5*, *RELA*, and *INHBA* as a strong candidate biomarker for predicting response to the detoxification approach. The reversal of alcohol-induced dysregulation in genes related to synaptic plasticity and the shift in gene–gene interactions suggest that the therapy facilitates recovery at the molecular level. Several genes that were upregulated or downregulated due to alcohol exposure showed a significant reversal towards the expression levels seen in healthy controls. This outcome suggests that the intervention effectively alleviates some of the molecular disruptions caused by alcohol use. Additionally, the reduction in the number of strong correlations (with rho values > 0.8 or < −0.8) from 355 before treatment to 196 after treatment reflects a shift towards gene expression patterns more similar to those of healthy controls. This shift suggests that the gene expression network may be returning to a more regulated state, less influenced by the chaotic expression patterns induced by alcohol consumption. These observations highlighted in our results are compatible with the current literature that shows an early effect of alcohol withdrawal towards the improvement of neuropsychological impairments [82].

## 4. Materials and Methods

### 4.1. Participants

A prospective, observational study of adult patients follow-up at First Department of Psychiatry of Aeginition University Hospital, in Greece, was performed. Twenty patients (17 men and 3 women, mean age 52 ± 6.93 years) fulfilled the DSM-V diagnostic criteria for alcohol abuse/dependence [83] and were admitted at this specialized department for alcohol detoxification on an inpatient basis. Subjects included in this study had to fulfill the following criteria: (a) age between 18 and 70 years, (b) absence of serious physical illness (as assessed through physical examination and routine laboratory screening), (c) absence of another pre- or co-existing major psychiatric disorder on the DSM-V, (d) absence of another drug abuse, and (e) the mere presence of affective symptoms or symptoms of anxiety was not considered to be an exclusion criterion. Ten healthy individuals (7 men and 3 women, mean age 48 ± 5.23 years) were included as controls. Participation in this project was on a voluntary basis. This study was approved by the hospitals’ institutional review board (346/19-4-22), and all patients provided their informed consent to participate. Subjects were treated on an inpatient basis and had to abstain from alcohol. Before the start of therapy and after 12 months, peripheral blood samples were obtained and stored at −80 °C until use.

### 4.2. Participant Evaluation and Response Characterization

A general clinical and biochemical medical screen was performed to exclude severely impaired individuals. Participants were diagnosed by the Schedules for Clinical Assessment in Neuropsychiatry and assessed through the Composite International Diagnostic Interview [84] (section on alcohol consumption) for their pattern of alcohol abuse, potential major life problems related to alcohol consumption, and the occurrence of withdrawal symptoms in the past; a structured questionnaire similar to the one proposed by the World Health Organization [85] was also used to assess the pattern of alcohol use. This questionnaire included items related to lifetime, past year, and past month frequency and quantity of alcohol use. Furthermore, sociodemographic data (age, socioeconomic status, marital status, level of education) and previous psychiatric history (pre-existent diagnosis, medication, hospitalizations) were recorded.

The WAIS-IV (Wechsler Adult Intelligence Scale-Fourth Edition) and the MMPI (Minnesota Multiphasic Personality Inventory) are two examples of neuropsychological exams that are used to generate tailored treatment plans. This action helps to ensure that underlying psychological problems are addressed in a complete manner [86,87].

A standard detoxification protocol was applied, comprising a short-term psychotherapy of cognitive–behavioral orientation. Psychotherapeutic treatment consisted both of individual sessions (PSAI) and family interventions. Vitamin replacement was initiated (Vitamin C, E, Vitamins of the B complex) and oral diazepam was administered (30–60 mg per day) upon intake. Diazepam was gradually tapered off, with a reduction rate of 20% every 2 days and was stopped after 7–10 days. No other medication was used over this period. Additionally, a meta-cognitive stress management technique that aims to reduce stress and improve physical and mental health, the Pythagorean Self Awareness Intervention (PSAI), was also used. It involves a cognitive restructuring based on the principles of Pythagoras’ philosophy, which activates the process of a deep spiritual introspection and allows the trainee to evaluate, reward, and criticize his actions independently of the toxic effects of the stressful event and the negative emotion that distorts thinking and life choices. The specific program treats individuals holistically; individuals come into full contact with themselves, evaluating their actions through self-observation. Via this path, they detect any automatic thoughts, rationalize them, and finally replace them with more functional ones. This metacognitive technique influences the cognitive triad of thought–emotion–behavior, since, through recall and self-observation, compunctions and ruminations are reduced situations that foster the development of psychopathology. PSAI seems to activate the circuit of introspection (“inner consciousness”), a neurobiological process that relates with Default Mode Network (DMN). The DMN is a neurological connection system regarding cognitions that emanates from self-relevant procedures and thereby contributes to corrective behaviors of the individual based on higher cognitive functions. Apart from the cognitive level, PSAI changes and affects the whole lifestyle and the daily routine of the participant, a result with great importance in addiction issues [88].

In research on alcohol use disorder (AUD), definitions of relapse are notably inconsistent and contentious. Defining relapse is essential for comprehending AUD recovery trajectories and the impact of specific events, such as a return to drinking, on clinical outcomes, including microbiome alterations. Definitions of relapse in the literature range from “any alcohol consumption” to “episodes of heavy drinking” [89]. Incorporating both remission and the biopsychosocial improvements into the definition of relapse seems crucial [90]. The DSM-5 defines AUD remission as a period in which no symptoms (excluding cravings) are present [83]. Relapse can be regarded as either a conclusion (complete return to excessive drinking) or a component of a dynamic transformation process, wherein lapses frequently occur but do not invariably result in a full relapse [91,92].

It is essential for our study to choose a relapse definition that harmonizes clinical significance with feasibility. We recommend employing the DSM-5 AUD diagnostic criteria during the follow-up or the threshold of 4/5 drinks for women/men after 4 days of abstinence, as endorsed by NIAAA (2005) guidelines [93] and utilized in treatment trials [91]. This term corresponds with the notion of “heavy drinking” as an indicator of relapse, which has been linked to adverse psychosocial consequences and diminished recovery pathways [92,93,94]. Furthermore, characterizing relapse as the intake of 4 to 5 alcoholic beverages enables the assessment of significant alterations in alcohol consumption that are likely to influence the microbiota, so offering a more refined comprehension of relapse is beneficial in relation to both biological and clinical consequences. This method resolves the ambiguity in relapse definitions by concentrating on a quantifiable outcome that indicates substantial alterations in alcohol use and the related physiological and psychological hazards [89,90]. This definition also facilitates the monitoring of microbiota alterations after substantial drinking episodes, yielding crucial insights into the relationship between relapse and gut health over time. With these criteria, 14 patients were characterized as responders and 6 as non-responders.

### 4.3. Cohort Stratification

For the purposes of our analyses, our samples were grouped in the following categories and were then pairwise analyzed: group 1 are alcoholics who responded to our intervention at baseline, group 2 are alcoholics who did not respond to our intervention at baseline, group 3 are the same subjects of group 1 after therapy, group 4 are the same subjects of group 2 after therapy, and, finally, we have the group of our non-alcoholic control subjects. We compared all samples of alcoholics at baseline with our healthy controls to profile gene expression changes associated with alcohol abuse. Next, we compared responders’ to non-responders’ expression profiles at baseline to identify transcriptional differences, which could serve as predictive factors between individuals who would and would not respond to therapy, given that the outcome is known. Finally, we compared the transcriptomic differences imposed by our intervention to assess its effects upon success.

### 4.4. Differential Gene Expression Analysis (DGEA)

Total RNA from peripheral blood was extracted using the Qiagen AllPrep RNA/DNA Mini Kit (Qiagen, Hilden, Germany). cDNA synthesis was performed with the RT2 First Strand Kit (Qiagen) following the manufacturer’s guidelines. Gene expression was quantified using the RT2 Profiler for Human Synaptic Plasticity RT-PCR Array (PAHS-126ZA, Qiagen) with the RT2 qPCR SYBR Green Master Mix (Qiagen), testing a total of 84 genes.

Differential gene expression analysis (DGEA) was performed using the RT2 Profiler PCR Array Data Analysis version 3.5 software from Qiagen on the following group pairs: (1) group 1 and group 2 samples together versus controls to determine gene expression changes profiling alcohol abuse, (2) group 1 versus group 2 samples to identify specific differences at baseline between those individuals who will and will not respond to therapeutic intervention, and, finally, (3) group 3 versus group 1 to identify the effects of a successful therapeutic intervention. Unfortunately, group 4 did not have enough samples to allow us to perform DGEA. All samples satisfied the quality criteria for PCR Array reproducibility, RT efficiency, and the absence of genomic DNA contamination.

Using five housekeeping genes (*ACTB*, *B2M*, *GAPDH*, *HPRT1*, and *RPLP0*) for within-sample normalization, we calculated the 2^−ΔCt^ values for all our genes. Fold change differences were determined by the 2^−ΔΔCt^ method and were reported in the form of fold regulation in the results (genes exhibiting under-expression are denoted as the negative reciprocal of the fold change, while overexpressed genes are presented as the fold change). Statistical significance was assessed using Student’s *t*-tests conducted on the 2^−ΔCt^ values for each gene in all group comparisons. All results were deemed as statistically significant based on their *p* < 0.05 values and their fold regulation thresholds of less than −2 or greater than 2. Plots were created in R v. 4.3.0 [95], and log_2_(1/ΔCt) values were used to normalize and visualize expression differences using the ggplot2 [96] package.

### 4.5. Participant Evaluation and Response Characterization

Correlation analysis is important for deciphering complex biological systems and identifying potential relationships between genes. By investigating what are essentially co-expression patterns, we can uncover the regulatory mechanisms underlying various biological processes and highlight genes that show coordinated expression changes across different conditions. To complement our DGEA and explore how the dysregulated genes might interact with each other, we employed an R once again. We calculated Spearman’s correlation coefficient (rho) for each gene pair and constructed a correlation matrix using the cor() function for each group used in our DGEA. We chose Spearman’s non-parametric test, as the Shapiro–Wilk normality test indicated that gene expressions were not all normally distributed. The correlation matrix was then filtered to include only gene pairs with rho values less than −0.8 (inverse correlation, when one gene is upregulated, the other is downregulated and vice versa) or greater than 0.8, thereby capturing only the strongest relationships across all the samples under study.

## 5. Conclusions

In conclusion, our study combined blood expression profiles that, when detected before therapy, may predict response to alcohol detoxification approaches. Collectively, our results set the background for both accomplishing significant insights into synaptic pathways in AUD and also developing predictors of detoxification therapy failure. Additionally, our study supports that the expression pattern of genes related to synaptic plasticity in peripheral blood can be a novel tool for clinical diagnosis, prognostication, and monitoring therapeutic responses by a minimally invasive approach. Validation of our findings in larger patient cohorts will be essential before the further development of such biomarkers is proposed.

## Data Availability

The data generated and/or analyzed during the current study are available from the corresponding author upon reasonable request.
